# Shared antibiofilm targets of biofilm regulators Wor3 and Bcr1 in *Candida albicans*

**DOI:** 10.1093/genetics/iyag129

**Published:** 2026-05-22

**Authors:** Katharina Goerlich, Norma V Solis, Scott G Filler, Aaron P Mitchell

**Affiliations:** Department of Microbiology, University of Georgia, Athens, GA 30602, United States; Division of Infectious Diseases, Lundquist Institute for Biomedical Innovation at Harbor-UCLA Medical Center, Torrance, CA 90502, United States; Division of Infectious Diseases, Lundquist Institute for Biomedical Innovation at Harbor-UCLA Medical Center, Torrance, CA 90502, United States; David Geffen School of Medicine, University of California, Los Angeles, Los Angeles, CA 90095, United States; Department of Microbiology, University of Georgia, Athens, GA 30602, United States

**Keywords:** *Candida albicans*, biofilm formation, antibiofilm genes, transcriptional regulation

## Abstract

*Candida albicans* is an opportunistic fungal pathogen and a component of the human microbiome. *C. albicans* virulence traits include biofilm production, which is governed by a large transcriptional network. Mutations of some biofilm regulators cause the same severe biofilm-defective phenotype in multiple clinical isolates. Mutations of others, such as Wor3, Bcr1, Ndt80, and Ume6, have mild or variable phenotypes among clinical isolates. We hypothesized that Wor3 may share functions with another variable-phenotype biofilm regulator. This hypothesis predicts that a double mutant lacking Wor3 and the shared-function regulator will have a severe biofilm defect in all clinical isolates. We observed that a *wor3*Δ/Δ *bcr1*Δ/Δ double mutant has a severe biofilm defect in vitro in 5 strain backgrounds tested. It also has a severe oral biofilm defect in a mouse oropharyngeal candidiasis model in the SC5314 strain background. RNA-seq data indicate that 5 genes encoding cell surface/secreted proteins are upregulated in *wor3*Δ/Δ, *bcr1*Δ/Δ, and *wor3*Δ/Δ *bcr1*Δ/Δ strains: *CWH8, DAG7, JEN2, PGA6,* and *YWP1.* Deletion mutations of *CWH8, DAG7, PGA6,* or *YWP1* enable biofilm formation in vitro in an SC5314-derived *wor3*Δ/Δ *bcr1*Δ/Δ strain, and deletion of *YWP1* enables biofilm formation in vitro in *wor3*Δ/Δ *bcr1*Δ/Δ strains from 4 other genetic backgrounds. *YWP1* has been shown to have antibiofilm activity previously, but *CWH8, DAG7,* and *PGA6* are newly described antibiofilm genes. Our study illustrates the value of strain variation considerations for gene function analysis and the importance of repression targets of biofilm regulators. In addition, our results expand the number of antibiofilm genes.

## Introduction

Biofilm formation has a central role in microbial infection biology (Sauer et al. 2022; Ramage et al. 2025). Biofilm communities persist on host tissues and medical devices, have increased resistance to antimicrobials, and protect cells from host immune clearance. Biofilms also release free-living planktonic cells, which can disseminate to infect additional sites. These considerations have driven the study of pathogenic biofilms for over 40 years (Costerton et al. 1987).

Our focus is biofilm formation by the fungal commensal and pathogen *Candida albicans* (Ramage et al. 2025). Biofilm-based infections by *Candida* species encompass a wide range of clinical scenarios, affecting diverse body sites and patient populations (Ramage et al. 2023). *C. albicans* is the major *Candida* pathogen in most settings (Ge and Wang 2022; Day and Sobel 2025; Schroeder et al. 2025), which is the reason we focus on this species. Our goal is to define major genetic determinants of biofilm formation to understand the process and relevant therapeutic targets.

Biofilm development by *C. albicans* depends upon over 150 genes, and perhaps as many as 1,000 (Nobile and Johnson 2015). Expression of many biofilm-related genes is under control of a set of master regulators (Nobile and Johnson 2015). The master regulators are transcription factors for which mutations cause pronounced biofilm aberrations in a variety of in vitro and in vivo conditions (Nobile et al. 2012; Fox et al. 2015). They also bind to one another's promoter regions and, in some cases, are required for one another's expression (Nobile et al. 2012; Fox et al. 2015). Some master regulators act through formation of phase-separated complexes with one another (Frazer et al. 2020; Ganser et al. 2023), a finding that helps explain how they bind to many of the same genomic regions (Nobile et al. 2012; Fox et al. 2015).

Our understanding of *C. albicans* biofilm determinants comes primarily from studies of the reference strain SC5314 and its marked derivatives. Use of this strain set simplifies comparison of results from different labs, and supports applicability of protocols for genetic manipulation and analysis. However, availability of genome sequences for hundreds of *C. albicans* isolates now facilitates genetic manipulation and genomic comparisons among diverse isolates (Anderson and Dietz 2024; Lindemann-Perez and Perez 2024). Sequence and ploidy variation has enabled discovery of new genes that govern pathogenicity, commensalism, and drug susceptibility (Anderson and Dietz 2024; Lindemann-Perez and Perez 2024). The availability of characterized diverse isolates has also prompted genotype-phenotype analysis, which makes an appraisal of effects of genetic background on the phenotype associated with a specific mutation (Dowell et al. 2010; Albert and Kruglyak 2015; Schacherer 2016; Sardi and Gasch 2018). We can conceptualize background effects on phenotype as arising from genetic modifiers in each background that shape mutational impact (Dowell et al. 2010; Albert and Kruglyak 2015; Schacherer 2016; Sardi and Gasch 2018). Among many applications of this type of analysis, our main goals have been (i) to determine whether a mutant phenotype is uniform and robust among clinical isolates, and (ii) to use the concept of genetic modifiers to shape our approaches to genetic analysis.

Our initial studies showed that some biofilm regulators, such as Efg1 and Brg1, have quite uniform phenotypic impact (Huang et al. 2019). In other words, a deletion mutation affecting each regulatory gene (*efg1*Δ/Δ or *brg1*Δ/Δ) causes the same severe biofilm-defective phenotype in multiple clinical isolates. Other biofilm regulators, such as Bcr1 and Ume6, have variable impact (Huang et al. 2019). We observed that modest differences in activity of interacting genes could reshape the relationship between a transcription factor and its target genes (Do et al. 2022). Such modest differences were frequent in comparisons between isolates (Huang et al. 2019; Do et al. 2022; Cravener et al. 2023), and thus help to explain the observation of genotype-phenotype variation, in keeping with studies of many other organisms (Hartman et al. 2001; Dowell et al. 2010; Chow 2016).

The study we present here began with the puzzle of why the transcription factor Wor3 has a mild and variable-phenotype among clinical isolates. Wor3 was implicated as a biofilm regulator because its expression is activated by master regulator Efg1 in 17 different clinical isolates (Cravener et al. 2023). In fact, increased *WOR3* expression could overcome the severe biofilm defect of an *efg1*Δ/Δ mutant, an observation that suggested that Wor3 has a major role in biofilm formation (Cravener et al. 2023). But, if so, we expect a *wor3*Δ/Δ mutant to have a uniformly severe biofilm defect, which it does not. Here we follow the logic of synthetic interaction analysis (Mackay 2014; Botstein 2015) to define functional redundancy between Wor3 and Bcr1. We use that understanding to define a group of target genes shared by Wor3 and Bcr1 that have antibiofilm functions. Our study provides a simple framework for analysis of mutations that have variable or strain-dependent impact on pathogenicity traits, and emphasizes the significance of negative control by biofilm regulators.

## Materials and methods

### Strains and media

C. *albicans* strains SC5314, P76067, P57055, P87, and P75010 and their derived *his*Δ/Δ mutants were used for transformations (Supplementary Table 1) (Huang et al. 2019). All strains were stored as glycerol stocks at −80 °C. Strains were streaked out 2 days before use at 30 °C on YPD (2% Bacto peptone, 2% dextrose, 2% agar and 1% yeast extract) and then cultured overnight at 30 °C in YPD liquid medium (2% Bacto peptone, 2% dextrose, and 1% yeast extract). All *C. albicans* transformants were selected on the following plates (2% agar): CSM-HIS (0.67% yeast nitrogen base without amino acids, 0.079% CSM-HIS, and 2% dextrose), YPD+NAT (2% Bacto peptone, 2% dextrose, 2% agar and 1% yeast extract+ 400 μg/mL nourseothricin [clonNAT, Gold Biotechnology]), YPD+HYGB (2% Bacto peptone, 2% dextrose, and 1% yeast extract, 600 μg/mL Hygromycin and 1.75 mg/mL Quinine [Park et al. 2022]), and YPD+KAN (2% Bacto peptone, 2% dextrose, and 1% yeast extract, 2 mg/mL G418 and 1 mg/mL Molybdate [Park et al. 2022]). Liquid RPMI-1640 medium (Sigma-Aldrich Inc., St. louis) adjusted to pH 7.4 and supplemented with or without 10% FBS (Atlanta Biologicals Inc., Flower Branch) was used for biofilm formation, filamentation, and RNA cell cultures. All strains used in this study and their genotypes, as well as primers and plasmids, are listed in Supplementary Table 1.

### Mutant strain construction

Transformations were conducted in *C. albicans* as described previously (Vyas et al. 2015; Min et al. 2016). Transformations used the lithium acetate method (Min et al. 2016) or electroporation (Grahl et al. 2017) as described in Supplementary Table 1 in column “mode of transformation”. Homozygous knockout mutants were made by targeting each respective gene, which we refer to generically as “*YFG1* (Your Favorite Gene 1)” with 1 μg Cas9 DNA cassette, 1 μg sgRNA DNA cassette, 3 μg of *YFG1* deletion cassette. Plasmid pV1093 and primers “CaCas9/F” and “CaCas9/R” were used to amplify Cas9. (Please see Supplementary Table 1 for details about plasmids and primers, each in a separate tab.) The single guides were amplified using split-joint PCR with pV1093 and round 1 primers “YFG1 sgRNA-_/F”, “sgRNA/R”, “YFG1 SNR52-_/R”, “SNR52/F”, and round 2 using round 1 products, and round 3 utilizes “sgRNA/N” and “SNR52/N” to amplify the full single guide cassette. The deletion cassettes were constructed by amplifying the plasmid containing the desired marker with DC primers with the corresponding flanking regions and 80 base pairs of homology with *YFG1* (Supplementary Table 1). All genotypes were verified via PCR amplification of the inserted construct and native locus from genomic DNA of isolated transformant colonies. For double and triple mutant construction each modified ORF was reconfirmed for correct genotype after each transformation.

Complementation was achieved in 2 different ways. For the *wor3*Δ/Δ+*WOR3* strain the 9,000 base pair native *WOR3* promoter-*WOR3* ORF PCR constructed was made in 3 fragments. One, WOR3p-mdr1 F and internal overlap wor3p R, 2, internal overlap wor3p F and WOR3 P-pNAT R, and 3, pNAT for adp/F and pNAT 3′ R → MDR1 adap/R. This was transformed into the SC5314 *wor3*Δ/Δ mutant (MC501) and verified via PCR. For the *wor3*Δ/Δ*bcr1*Δ/Δ*ywp1*Δ/Δ triple mutant (KG90) complementation was achieved by using the *YWP1* promoter-*YWP1* ORF PCR construct. YWP1_Farupstream_F for MDR1_F and YWP1 3′ → kan 5′R was amplified as fragment 1 and KAN for adap/F and Kan 3′R → MDR1 adap/R for fragment 2. The transformants were verified using PCR.

### Biofilm assays

Strains were grown in 5 mL of liquid YPD for 18 h at 30 °C. RPMI was prewarmed at 37 °C and then 100 μL aliquoted into each well (Greiner 96 wells Cat#655090). Wells were then inoculated to a final OD_600_ of 0.05 and incubated at 37 °C for 90 min. After wells were washed with 1× PBS to remove any nonadherent cells and 100 μL of fresh RPMI media was added to each well. Plates were incubated at 37 °C for an additional 24 h at 60 RPM shaking. Supernatant was removed, and biofilms were washed again with 1× PBS. Biofilms were fixed with 100 μL of 4% formaldehyde in 1× PBS and incubated at room temperature for 1 h. Biofilms were washed once more in 1× PBS and stained overnight using 5.5 mg/mL of calcofluor white in 1× PBS. The next day biofilms were washed with 1× PBS and clarified using a 50% thiodiethanol and 50% 1× PBS for 1 h. This was followed by a 1-h incubation with 100% thiodiethanol. Clarified biofilms were imaged on a Keyence fluorescence microscope using PlanFluor 20× 0.45/8.80–7.5 mm Ph1 objective with 2× digital zoom. Technical replicates (*n* = 3 or more) of the biofilms were imaged within the wells and apical navigation images were taken to ensure even sampling. Each isolate has been replicated in at least 3 independent biofilm assays conducted on different days. Images were processed using Fiji software and all statistical analyses were carried out using GraphPad Prism.

### Filamentation assays

Cells were grown in 5 mL of liquid YPD rotating for 18 h at 30 °C. Prewarmed 5 mL of RPMI were inoculated to an OD600 of 0.5 from the overnight cultures and incubated for 4 h at 37 °C at 60 RPM. Cells were collected via centrifugation (2,800 RPM for 5 min) and fixed in 4% formaldehyde in 1× PBS for 15 min. Following, the samples were washed 1 time in 1× PBS and then stained using calcofluor white for at least 30 min and treated with Proteinase-K in a 37 °C water bath for 90 min. Cells were imaged on a Keyence fluorescence microscope using PlanFluor 20× 0.45/8.80–7.50 mm Ph1 objective with 2× digital zoom. Images were taken in triplicate and analyzed in Fiji.

### RNA extraction and data analysis

Cells were grown in 5 mL of liquid YPD rotating at 30 °C for 18 h. The next day, cells were inoculated to 25 mL of prewarmed RPMI-1640 media (Sigma-Aldrich, Inc., St. Louis) adjusted to pH 7.4 with or without 10% fetal bovine serum (Atlanta Biologicals Inc., Flower Branch) at an OD_600_ of 0.2. The cells were grown for 4 h at 225 RPM in a shaking incubator at 37 °C, then harvested by vacuum filtration and frozen at −80 °C until RNA extraction. Three biological replicates were provided for RNA-seq experiments.

RNA extraction was performed according to previously published method (Huang et al. 2019; Do et al. 2022). Cells were disrupted using Zirconia beads (Ambion, Fisher Scientific, Waltham), and extraction was performed using a 25:24:1 phenol:chloroform:isoamyl alcohol method combined with a Qiagen RNeasy Mini Kit (Qiagen, Venlo, Netherlands). RNA-Seq analysis and processing of raw fastq reads were performed by Novogene. Differential expression was assessed using DEseq2 (v 1.40.2) in R using alpha = 0.05.

### Oropharyngeal candidiasis model

Competition analysis in the oropharyngeal candidiasis model was performed by comparing colony-forming units between 2 *C. albicans* strains with distinct markers, as previously described (29). Briefly, male ICR mice were immunosuppressed with triamcinolone acetonide (40 mg/kg subcutaneously) 24 h prior to infection. Mice were sublingually inoculated with a 1:1 mixture of a mutant strain and a competitor (either SC5314 wild-type, *wor3*Δ/Δ*, bcr1*Δ/Δ, or *wor3*Δ/Δ *bcr1*Δ/Δ). On day 1 post-infection, tongue and oral tissues were excised, weighed, and homogenized. Homogenates were then plated on selective media, and CFUs were enumerated the following day.

### Data interpretation

Interpretations and hypotheses were informed by the extensive records available at the *Candida* Genome Database (Lew-Smith et al. 2025).

## Results

### Functional interactions with Wor3

Homozygous *wor3*Δ/Δ mutations produce a mild and variable biofilm defect in the strains SC5314 and P87 (Cravener et al. 2023). This observation may be explained by the idea that Wor3 shares its biofilm-related function with another transcription factor. The hypothetical second transcription factor may have low activity under conditions in which the *wor3*Δ/Δ phenotype is strong, and high activity under conditions in which the *wor3*Δ/Δ phenotype is weak. This idea predicts that mutational inactivation of the hypothetical transcription factor may also cause a mild and variable biofilm defect. It also predicts that a double mutant lacking both Wor3 and the second transcription factor will have a strong biofilm defect that is independent of strain background.

**Fig. 1.**
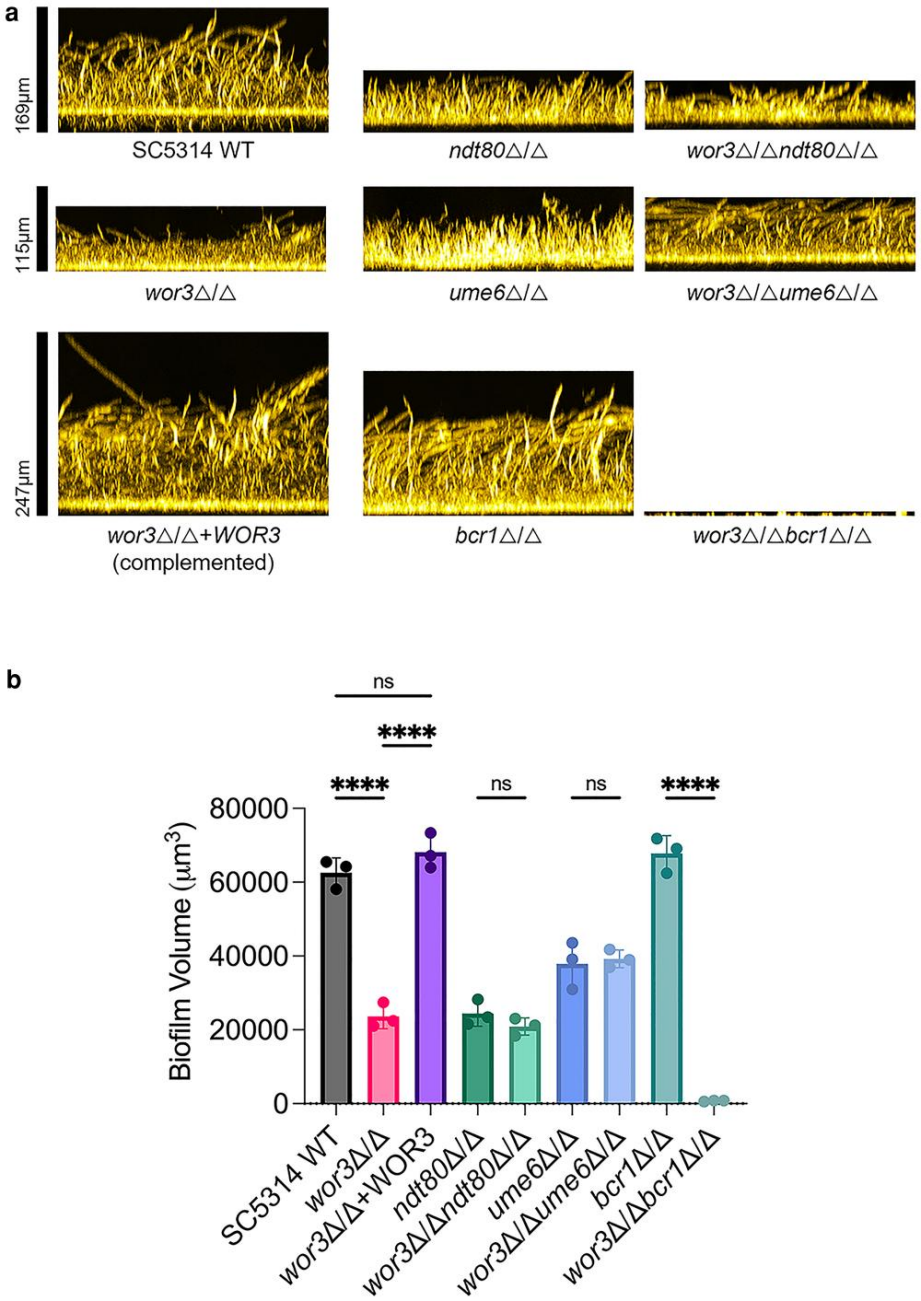
Biofilm assays of *C. albicans* SC5314-derived strains. Wild-type, *wor3*Δ/Δ+*WOR3* (complement), *wor3*Δ/Δ, *ndt80*Δ/Δ, *ume6*Δ/Δ, *bcr1*Δ/Δ, *wor3*Δ/Δ *ndt80*Δ/Δ, *wor3*Δ/Δ *ume6*Δ/Δ, and *wor3*Δ/Δ *bcr1*Δ/Δ strains, all in the SC5314 background, were assayed for biofilm formation in vitro. Strains were grown in RPMI medium in a 96 well plate at 37 °C for 24 h. Biofilms were fixed and stained with calcofluor white and imaged using a Keyence BZ-X800E fluorescence microscope. a) Representative biofilm 96 well plate side projection views. The scale bars 169 μm, 115 μm, and 247 μm, correspond to the first, second, and third row respectively. b) Biofilm volume measurements were obtained from 3 independent wells in a 96 well plate assay. A one-way ANOVA (Šídák's multiple comparisons test) test was used to determine statistical significance, as indicated by asterisks: * = ≤0.05; ** = ≤0.01; *** = ≤0.001; **** = ≤0.0001; ns = not significant.

Candidates for the hypothetical shared-function transcription factor include Ume6, Bcr1, and Ndt80. Deletion mutations of these candidates cause a mild and variable biofilm defect among different *C. albicans* strains (Huang et al. 2019; Goerlich and Mitchell 2026). We tested these candidates first through biofilm assays of single and double mutants in the SC5314 background. Biofilm formation was assayed in RPMI medium, which yields a mild *wor3*Δ/Δ biofilm defect (Cravener et al. 2023). Our assays showed that a *wor3*Δ/Δ mutant exhibited reduced biofilm depth and volume (Fig. 1a and b), and that complementation with *WOR3* expressed from its native promoter restored biofilm attributes that were comparable to the wild type (Fig. 1a and b). Based on biofilm depth and volume, candidate gene mutants *ndt80*Δ/Δ and *ume6*Δ/Δ had a modest biofilm defect, comparable to the *wor3*Δ/Δ mutant; candidate gene mutant *bcr1*Δ/Δ had no defect (Fig. 1a and b). Combining the *ndt80*Δ/Δ or *ume6*Δ/Δ mutations with a *wor3*Δ/Δ mutation yielded a biofilm defect that was similar to that of the *ndt80*Δ/Δ or *ume6*Δ/Δ single mutants (Fig. 1a and b). However, the *wor3*Δ/Δ *bcr1*Δ/Δ double mutant had a more severe biofilm defect than either the *bcr1*Δ/Δ or *wor3*Δ/Δ single mutants (Fig. 1a and b). This outcome suggests that Bcr1 and Wor3 share a biofilm-related function.

Filamentation defects evident in planktonic cells are often associated with biofilm defects (Nobile and Johnson 2015; Ramage et al. 2025). In planktonic filamentation assays, all strains described above produced filamentous cells, including the *wor3*Δ/Δ *bcr1*Δ/Δ double mutant (Supplementary Fig. 1). This observation suggests that a filamentation defect does not cause the biofilm defect of the *wor3*Δ/Δ *bcr1*Δ/Δ double mutant.

### Strain dependence of Wor3-Bcr1 functional interaction

**Fig. 2.**
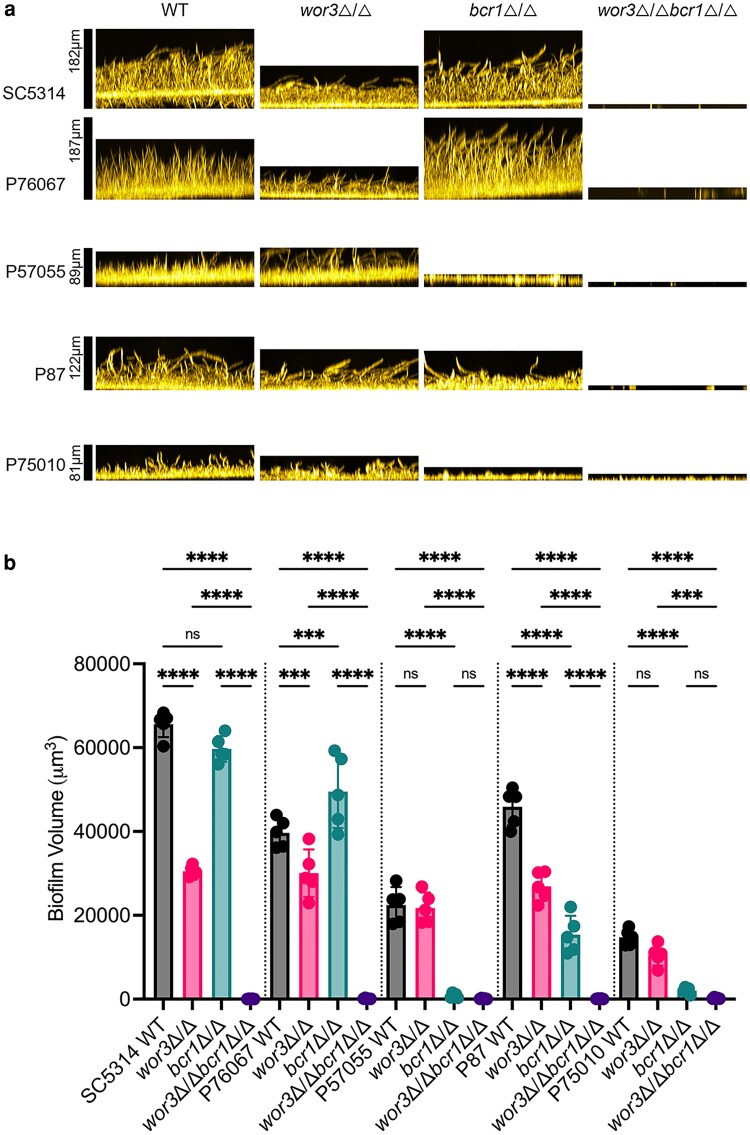
Biofilm assays in multiple strain backgrounds. *C. albicans* wild-type, *wor3*Δ/Δ, *bcr1*Δ/Δ, and *wor3*Δ/Δ *bcr1*Δ/Δ strains from the SC5314, P76067, P57055, P87, and P75010 backgrounds were assayed for biofilm formation in vitro. Strains were grown in RPMI medium in a 96 well plate at 37 °C for 24 h. Biofilms were fixed and stained with calcofluor white and imaged using a Keyence BZ-X800E fluorescence microscope. a) Representative biofilm 96 well plate side projection views. Scale bars: SC5314, 182 μm; P76067, 187 μm; P57055, 89 μm; P87, 122 μm; P75010, 81 μm. b) Biofilm volume measurements were obtained from 5 independent wells in a 96 well plate assay. A one-way ANOVA (Šídák's multiple comparisons test) test was used to determine statistical significance, as indicated by asterisks: * = ≤0.05; ** = ≤0.01; *** = ≤0.001; **** = ≤0.0001; ns = not significant.

**Fig. 3.**
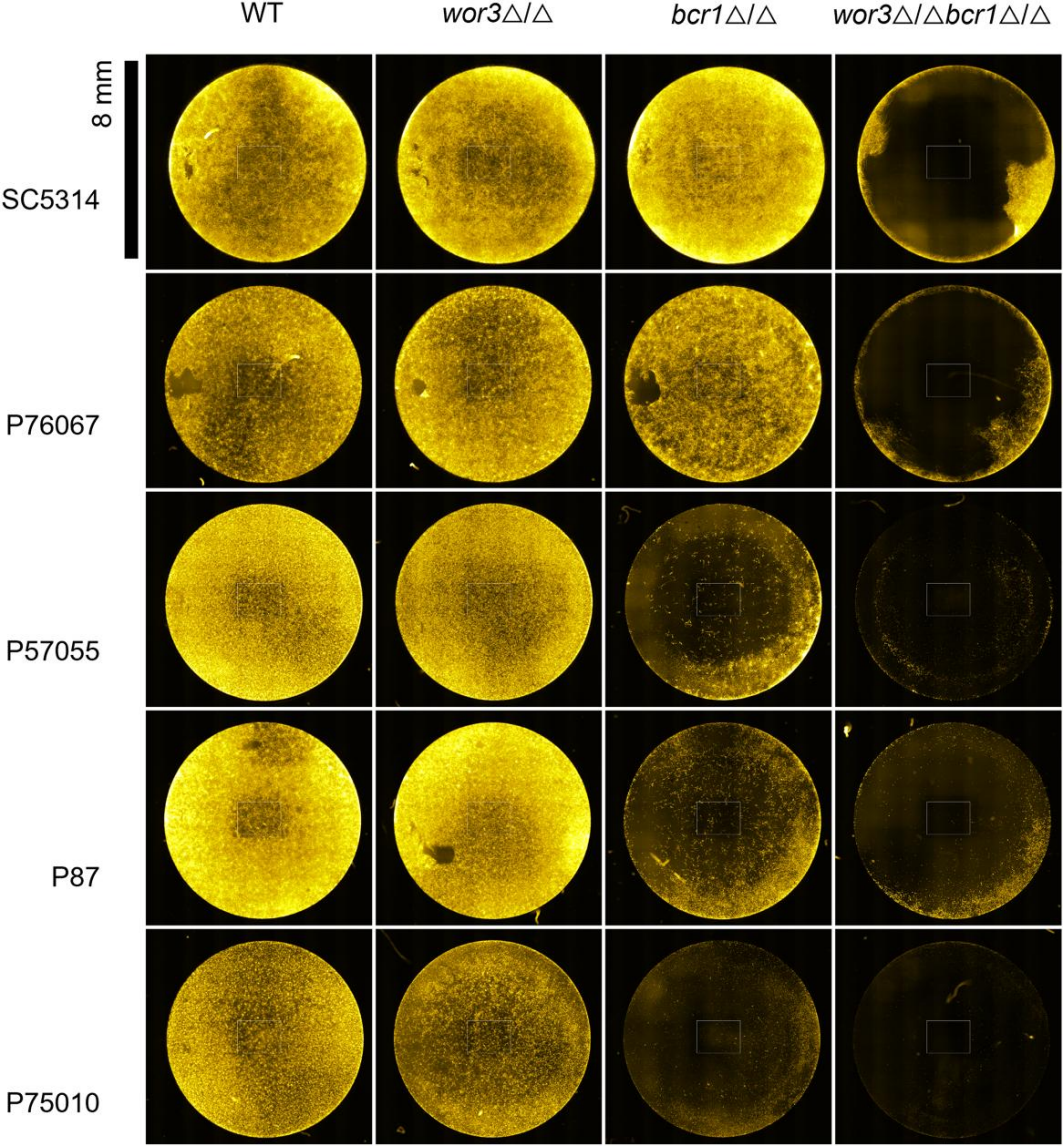
Representative biofilm 96 well plate apical navigation views. Images were obtained from 5 independent wells, and a representative image was chosen. These wells correlate to the side projection view biofilm images and biofilm volume measurements in Fig. 2. Each well is approximately 8 mm in diameter.

We sought to determine if the Wor3-Bcr1 functional interaction occurred in multiple strains. We used SC5314 and 4 additional clinical isolates used in previous strain variation studies (Huang et al. 2019; Cravener et al. 2023). These strains represent 5 major *C. albicans* clades: SC5314 (clade 1), P76067 (clade 2), P57055 (clade 3), P87 (clade 4) and P75010 (clade 11). We constructed single and double mutants of *WOR3* and *BCR1* in all backgrounds and assayed biofilm depth and volume in RPMI medium (Fig. 2a and b). The phenotypes of *wor3*Δ/Δ and *bcr1*Δ/Δ single mutants varied with genetic background, as expected. However, *wor3*Δ/Δ *bcr1*Δ/Δ double mutants presented a severe biofilm defect in all 5 backgrounds. For P57055 and P75010, the *bcr1*Δ/Δ single mutant alone had a severe biofilm defect. Therefore, the *wor3*Δ/Δ *bcr1*Δ/Δ double mutant defect was not statistically different from the *bcr1*Δ/Δ single mutant defect (Fig. 2b). However, navigation views of entire wells of the biofilm assay plates showed a qualitative difference: much of the surface seemed more sparsely populated by the *wor3*Δ/Δ *bcr1*Δ/Δ double mutants than by the *bcr1*Δ/Δ single mutant (Fig. 3). We conclude that a Wor3-Bcr1 functional interaction is evident in multiple strain backgrounds; it is statistically validated in 3 strains and qualitatively evident in the other 2. Moreover, a second feature of the data supports the shared-function hypothesis: in the 2 strains in which a *bcr1*Δ/Δ mutant has a severe biofilm defect (P87 and P75010), a *wor3*Δ/Δ mutant has no significant defect (Fig. 2b). This observation is consistent with the idea that the magnitude of impact of 1 transcription factor is inversely related to the magnitude of impact of the other.

### Wor3-Bcr1 functional interaction in an infection model

**Fig. 4.**
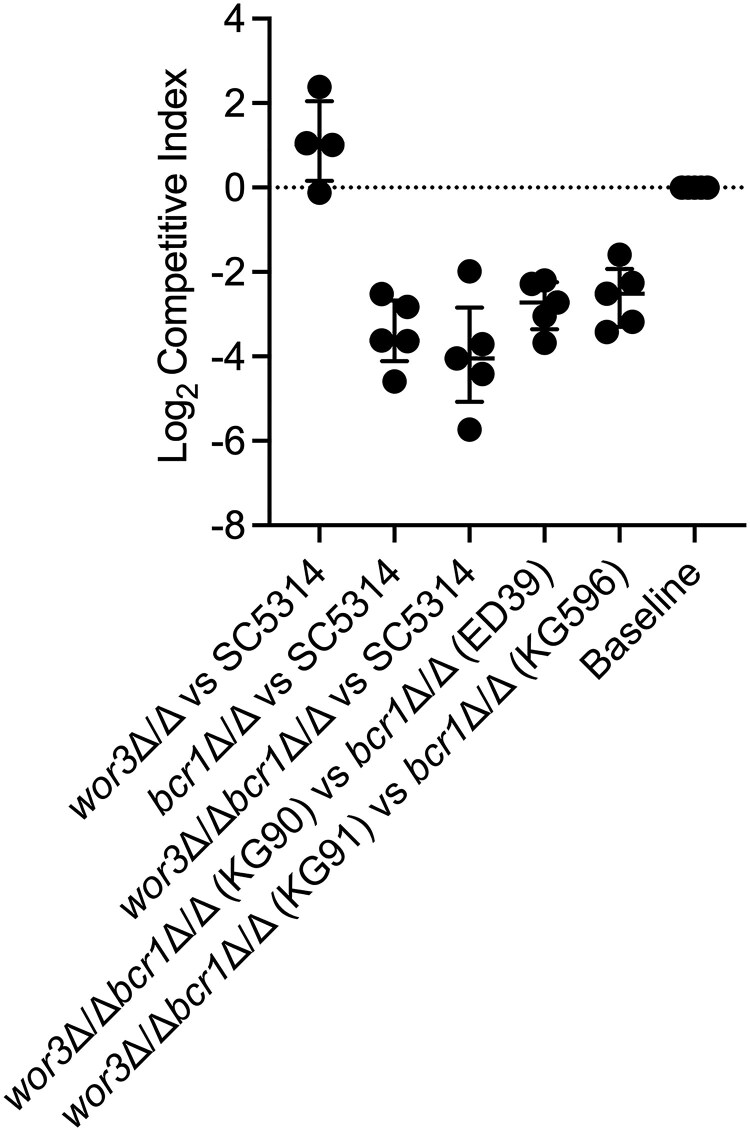
Oropharyngeal candidiasis assays. Competitive fitness was determined after sublingually inoculating immunosuppressed mice with a 1:1 mixture of the indicated strains. Tongue and oral tissues were harvested 24 h post-infection for CFU quantification. The competition index was calculated as the ratio of mutant to WT recovered from tissue. A competition index >1 indicates increased recovery of the first strain listed; a competitive index <1 indicated reduced recovery of the first strain listed. The *bcr1*Δ/Δ, and *wor3*Δ/Δ *bcr1*Δ/Δ were tested against wild type. Two independent *wor3*Δ/Δ *bcr1*Δ/Δ double mutants were also tested against 2 independent *bcr1*Δ/Δ mutants. The dashed baseline represents neutral competition, where neither strain has a fitness advantage over the other. Each point represents an individual mouse (*N* = 5 per group), and horizontal bars indicate the mean ± SD.

To test the significance of Wor3-Bcr1 functional interaction for pathogenicity, we turned to a mouse model of oropharyngeal candidiasis (Solis and Filler 2012), an in vivo biofilm infection (Ramage et al. 2025). Strains from the SC5314 background were tested through a competition paradigm in immunosuppressed mice. The animals were inoculated sublingually with a 1:1 mixture of SC5314 wild type and mutant strains, and the number of cells of each strain was measured 1 day later. Results are expressed as the ratio of recovered cells of each strain (Fig. 4). We observed that the *wor3*Δ/Δ single mutant had a slight competitive advantage (∼2-fold) compared with wild type, whereas the *bcr1*Δ/Δ single mutant and *wor3*Δ/Δ *bcr1*Δ/Δ double mutant had a competitive disadvantage (∼16-fold) compared with wild type. To test whether the *wor3*Δ/Δ *bcr1*Δ/Δ double mutant had a more severe defect than the *bcr1*Δ/Δ single mutant, we conducted competition assays between strains of those genotypes (Fig. 4). We used 2 independent *bcr1*Δ/Δ single mutants and 2 independent *wor3*Δ/Δ *bcr1*Δ/Δ double mutants. In each case, a *wor3*Δ/Δ *bcr1*Δ/Δ double mutant had a competitive disadvantage (∼8-fold) compared with a *bcr1*Δ/Δ single mutant. We conclude that a *wor3*Δ/Δ mutation exacerbates the *bcr1*Δ/Δ mutant defect in vivo in oropharyngeal infection. These outcomes argue that the Wor3-Bcr1 functional interaction is relevant to pathogenicity.

### Wor3 and Bcr1 functional target genes

**Fig. 5.**
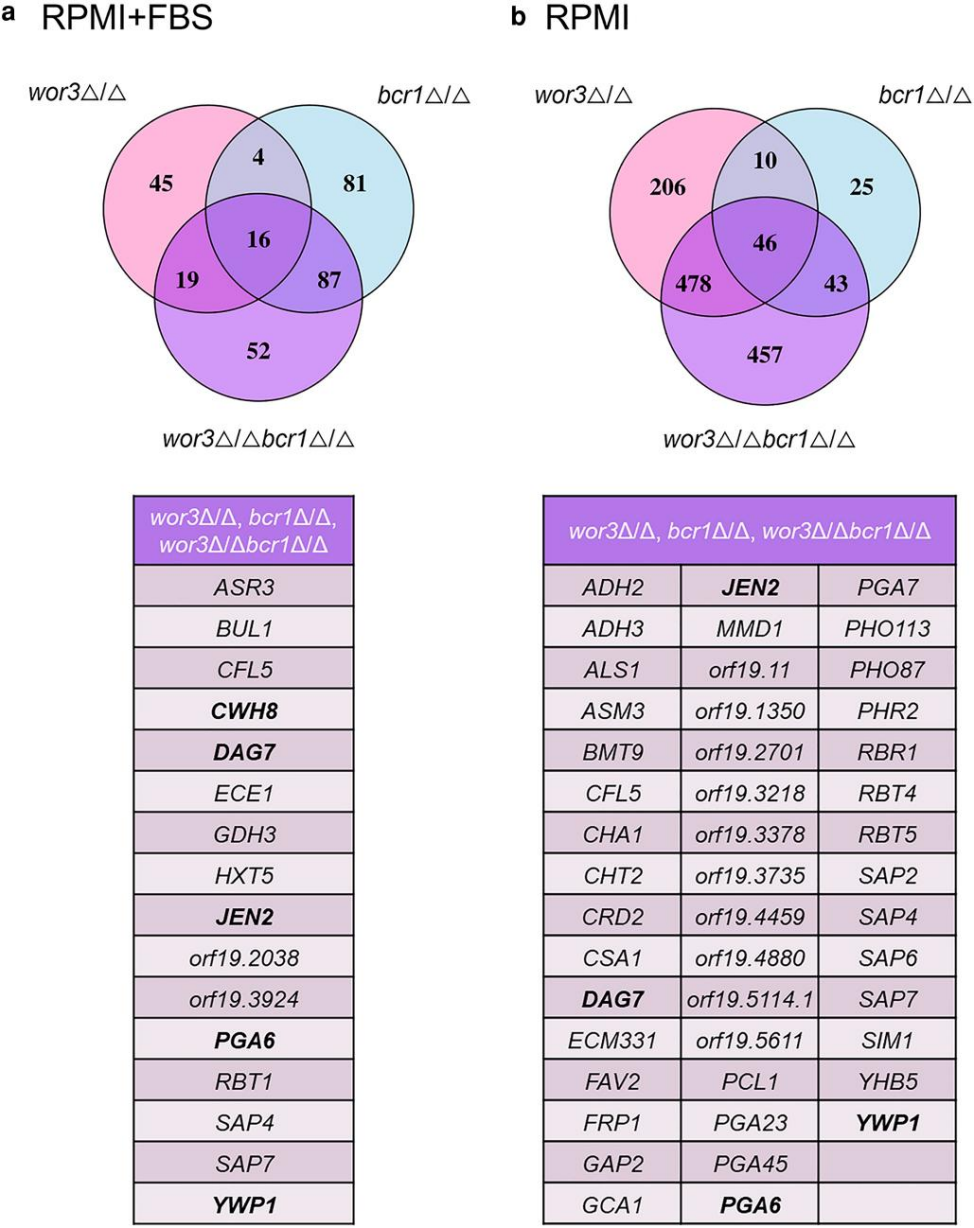
Gene expression analysis. RNA-sequencing (RNA-seq) was performed on 4 strains in the SC5314 background in 2 different media. SC5314, *wor3*Δ/Δ, *bcr1*Δ/Δ, and *wor3*Δ/Δ *bcr1*Δ/Δ strains were grown in either prewarmed RPMI+10% fetal bovine serum (RPMI+FBS) or prewarmed RPMI. Three independent cultures of each strain were used to prepare RNA, and RNA-seq was then performed. The entire data set is in Supplementary Table 2. a) Venn diagrams represent the genes that are significantly (adjusted *P*-value < 0.05) regulated with a Log_2_ fold-change >1 or <−1 in RPMI+FBS. The table below lists the 16 genes that were differentially expressed in all 3 backgrounds. b) Venn diagrams present the genes that are significantly (adjusted *P*-value < 0.05) regulated with a Log_2_ fold-change >1 or <−1 in RPMI. The table below lists the 46 genes that are differentially regulated in all 3 backgrounds. The genes in bold font (*CWH8, DAG7, JEN2, PGA6,* and *YWP1*) were selected for downstream functional analysis.

Our shared-function hypothesis predicts that Wor3 and Bcr1 will have downstream target genes in common, and that these target genes will impact biofilm formation. To identify such genes, we used RNA-seq analysis of SC5314-derived wild-type, *wor3*Δ/Δ, *bcr1*Δ/Δ, and *wor3*Δ/Δ *bcr1*Δ/Δ strains. Among many perspectives about how to identify functional targets, we chose to focus on genes whose RNA levels were significantly altered (*p*_adj_ < 0.05, |log_2_ Fold-Change| > 1) in each single mutant as well as the double mutant, each compared with the wild type. We conducted RNA-seq on samples grown in RPMI+10% fetal bovine serum (RPMI + FBS), which we have used extensively in the past, and in RPMI alone, the medium we used for phenotypic assays above. RPMI + FBS yield a synthetic *wor3*Δ/Δ *bcr1*Δ/Δ phenotype similar to that in RPMI (Supplementary Fig. 2). In RPMI + FBS, there were 174 genes differentially expressed in the *wor3*Δ/Δ *bcr1*Δ/Δ double mutant vs wild type, and only 16 of those genes were differentially expressed in both the *wor3*Δ/Δ vs wild type and *bcr1*Δ/Δ vs wild type comparisons (Fig. 5a). In RPMI, there were 1,024 genes differentially expressed in the *wor3*Δ/Δ *bcr1*Δ/Δ double mutant vs wild type, and only 46 of those genes were differentially expressed in both the *wor3*Δ/Δ vs wild type and *bcr1*Δ/Δ vs wild type comparisons (Fig. 5b). The adhesin gene *ALS1* was downregulated in *wor3*Δ/Δ *bcr1*Δ/Δ double mutant vs wild type comparison in RPMI + FBS (Fig. 5b); however, this gene expression change seemed unlikely to account for the *wor3*Δ/Δ *bcr1*Δ/Δ double mutant phenotype. The reason is that we have seen no impact of *ALS1* and the related adhesin gene *ALS3* on biofilm formation in RPMI medium (our unpublished results). Five other genes caught our attention—*CWH8, DAG7, JEN2, PGA6,* and *YWP1*—for 3 reasons. First, their protein products are all connected to the cell surface or extracellular region, which would be expected for proteins involved in biofilm adherence. Second, they were all significantly affected in the single and double mutants in RPMI + FBS, and 4 of the 5 were significantly affected in the single and double mutants in RPMI (with *CWH8* being the exception). These genes were upregulated in the *wor3*Δ/Δ *bcr1*Δ/Δ mutant, which would be consistent with a causal role in the double mutant biofilm defect. Finally, the cell surface and secreted protein Ywp1 has been characterized previously; in most (Granger et al. 2005; Sellam et al. 2009; Granger 2012; Granger 2018) though not all (McCall et al. 2019) studies it is associated with antiadhesion or antibiofilm activity. We reasoned that upregulation of antibiofilm functions in the *wor3*Δ/Δ *bcr1*Δ/Δ double mutant could be the cause of its biofilm defect.

**Fig. 6.**
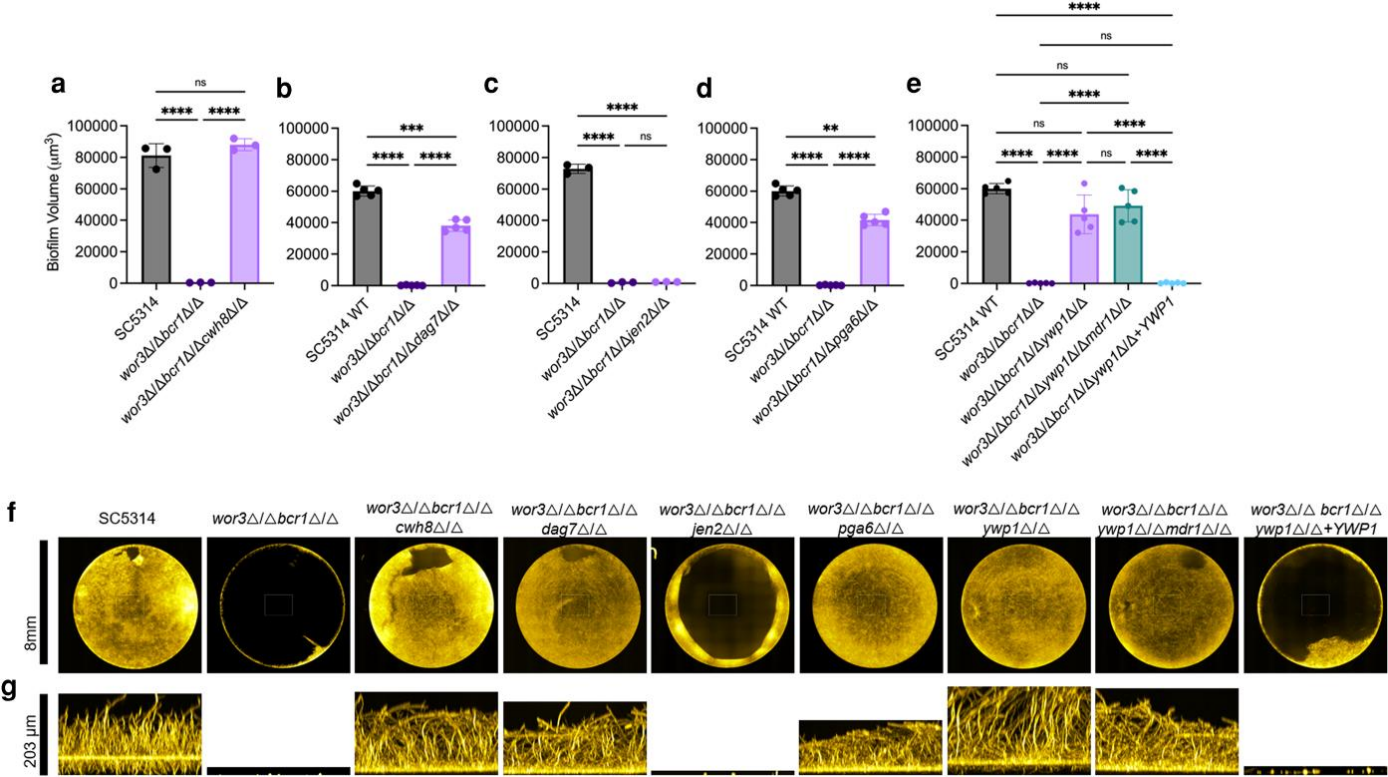
Biofilm assays. (a–e) Biofilm volume measurements in SC5314-derived strains. Strains were grown in RPMI medium in a 96 well plate at 37 °C for 24 h. Biofilms were fixed and stained with calcofluor white and imaged using a Keyence BZ-X800E fluorescence microscope. A one-way ANOVA (Šídák's multiple comparisons test) test was used to determine statistical significance, as indicated by asterisks: * = ≤0.05; ** = ≤0.01; *** = ≤0.001; **** = ≤0.0001; ns = not significant. a). Biofilm volume calculations from 3 independent wells for SC5314 wild-type, *wor3*Δ/Δ *bcr1*Δ/Δ, and *wor3*Δ/Δ *bcr1*Δ/Δ *cwh8*Δ/Δ strains. b). Biofilm volume calculations from 5 independent wells for SC5314 wild-type, *wor3*Δ/Δ *bcr1*Δ/Δ, and *wor3*Δ/Δ *bcr1*Δ/Δ *pga6*Δ/Δ strains. c). Biofilm volume calculations from 5 independent wells for SC5314 wild-type, *wor3*Δ/Δ *bcr1*Δ/Δ, and *wor3*Δ/Δ *bcr1*Δ/Δ *dag7*Δ/Δ strains. d). Biofilm volume calculations from 3 independent wells for SC5314 wild-type, *wor3*Δ/Δ *bcr1*Δ/Δ, and *wor3*Δ/Δ *bcr1*Δ/Δ *jen2*Δ/Δ strains. e). Biofilm volume calculations from 5 independent wells for SC5314 wild-type, *wor3*Δ/Δ *bcr1*Δ/Δ, *wor3*Δ/Δ *bcr1*Δ/Δ *ywp1*Δ/Δ, *wor3*Δ/Δ *bcr1*Δ/Δ *ywp1*Δ/Δ *mdr1*Δ/Δ, and *wor3*Δ/Δ *bcr1*Δ/Δ *ywp1*Δ/Δ *mdr1*Δ/Δ+*YWP1* strain backgrounds. f). Representative biofilm 96-well plate apical navigation views. Scale bar on the left is 8 mm. g). Representative biofilm 96-well plate side projection views. Scale bar on the left at 203 μm.

If the select target genes inhibit biofilm formation in the *wor3*Δ/Δ *bcr1*Δ/Δ double mutant, then deletion of those target genes should restore biofilm formation in the double mutant background. We constructed the relevant triple mutants and assayed biofilm depth and volume (Fig. 6). In the SC5314 *wor3*Δ/Δ *bcr1*Δ/Δ double mutant background, biofilm formation was significantly restored by the *cwh8*Δ/Δ, *dag7*Δ/Δ, *pga6*Δ/Δ, and *ywp1*Δ/Δ deletions (Fig. 6a, b, and d–g). There was no improvement of biofilm formation by the *jen2*Δ/Δ deletion (Fig. 6c, f, and g). These results support the idea that each of the genes *CWH8, DAG7, PGA6,* and *YWP1* has an antibiofilm function, and that their upregulation in the *wor3*Δ/Δ *bcr1*Δ/Δ double mutant causes a biofilm defect.

**Fig. 7.**
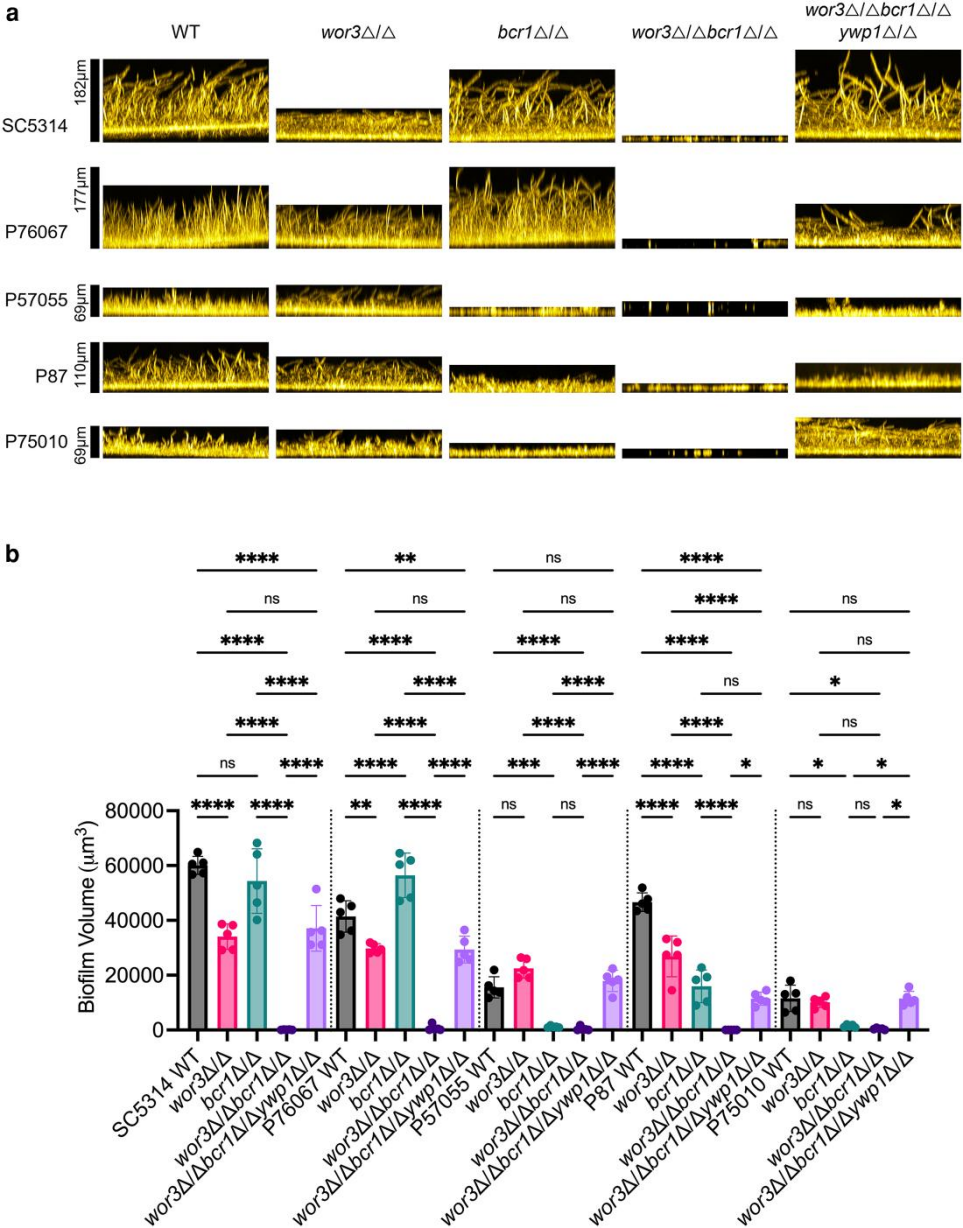
Biofilm assays for multiple strain backgrounds. *C. albicans* wild-type, *wor3*Δ/Δ, *bcr1*Δ/Δ, *wor3*Δ/Δ *bcr1*Δ/Δ *wor3*Δ/Δ *bcr1*Δ/Δ *ywp1*Δ/Δ strains from the SC5314, P76067, P57055, P87, and P75010 backgrounds were assayed for biofilm formation in vitro. Strains were grown in RPMI medium in a 96 well plate at 37 °C for 24 h. Biofilms were fixed and stained with calcofluor white and imaged using a Keyence BZ-X800E fluorescence microscope. a) Representative biofilm 96 well plate side projection views. Scale bars: SC5314, 182 μm; P76067, 177 μm; P57055, 69 μm; P87, 110 μm; P75010, 69 μm. b) Biofilm volume measurements were obtained from 5 independent wells in a 96 well plate assay. A one-way ANOVA (Šídák's multiple comparisons test) test was used to determine statistical significance, as indicated by asterisks: * = ≤0.05; ** = ≤0.01; *** = ≤0.001; **** = ≤0.0001; ns = not significant.

We further validated *YWP1* function in this system with 2 approaches. First, we complemented the *ywp1*Δ/Δ mutation. In the SC5314-derived *wor3*Δ/Δ *bcr1*Δ/Δ *ywp1*Δ/Δ triple mutant, we integrated a wild-type copy of *YWP1* at an ectopic site, the *MDR1* gene, to create a *wor3*Δ/Δ *bcr1*Δ/Δ *ywp1*Δ/Δ *mdr1*Δ::*YWP1/mdr1*Δ::*YWP1* strain. This strain was biofilm-defective, whereas a control *wor3*Δ/Δ *bcr1*Δ/Δ *ywp1*Δ/Δ *mdr1*Δ/Δ strain was not (Fig. 6e, f, and g). This result indicates that the *ywp1*Δ/Δ mutation, not a secondary mutation, is the cause of biofilm formation ability in the *wor3*Δ/Δ *bcr1*Δ/Δ *ywp1*Δ/Δ strain. Second, we constructed *wor3*Δ/Δ *bcr1*Δ/Δ *ywp1*Δ/Δ triple mutants in the 4 additional strain backgrounds used earlier. Biofilm formation was significantly improved in all *wor3*Δ/Δ *bcr1*Δ/Δ mutants by the *ywp1*Δ/Δ mutation (Fig. 7a and b). We conclude that *YWP1* expression contributes to the *wor3*Δ/Δ *bcr1*Δ/Δ biofilm defect in 5 of 5 *C. albicans* strain backgrounds.

## Discussion

**Fig. 8.**
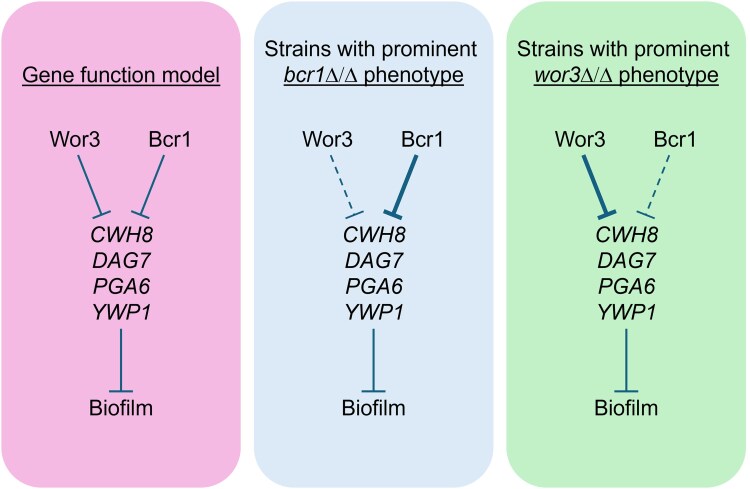
Relationships among Wor3, Bcr1, and antibiofilm genes. The gene function model (left panel) illustrates how Wor3 and Bcr1 are together required for biofilm formation. This conclusion comes from the finding that *wor3*Δ/Δ *bcr1*Δ/Δ double mutants are biofilm-defective in multiple *C. albicans* isolates. Wor3 and Bcr1 each negatively regulate the genes *CWH8, DAG7, PGA6,* and *YWP1*, and other genes. This conclusion comes from the observation that *CWH8, DAG7, PGA6,* and *YWP1* are all upregulated in *wor3*Δ/Δ, *bcr1*Δ/Δ, and *wor3*Δ/Δ *bcr1*Δ/Δ mutants under either or both RPMI and RPMI+FBS growth conditions. The *CWH8, DAG7, PGA6,* and *YWP1* gene products are known or predicted to be cell surface or secreted proteins. Each of the 4 genes functions as antibiofilm genes, because deletion of any one in a *wor3*Δ/Δ *bcr1*Δ/Δ strain enables biofilm formation. We explain the observation that some strains have a prominent *bcr1*Δ/Δ biofilm defect and a modest *wor3*Δ/Δ biofilm defect (central panel) with the proposal that Bcr1-dependent repression is more active than Wor3-dependent repression in those strains. Strains P57055 and P75010 present such biofilm phenotypes. We explain the observation that some strains have a modest *bcr1*Δ/Δ biofilm defect and a prominent *wor3*Δ/Δ biofilm defect (right panel) with the proposal that Bcr1-dependent repression is less active than Wor3-dependent repression in those strains. Strains SC5314 and P76067 present such biofilm phenotypes.

Biofilm formation ability supports both commensalism and pathogenicity (Ramage et al. 2023; Ramage et al. 2025). For almost all microbes, biofilm growth increases resistance to drugs and environmental perturbations (Sauer et al. 2022). For *C. albicans* and many opportunistic pathogens, biofilm-related infection rates continue to increase because of complex medical interventions (Ramage et al. 2025). Our ability to eradicate pathogenic biofilms is informed by understanding the gene products that govern biofilm formation and the extent of their impact among diverse strains and species. In this study we have contributed to that effort in 2 ways. First, we used observations of strain variation to hypothesize functional relationships, and validated the hypothesis with *WOR3* and *BCR1.* The concept of synthetic genetic interaction is well-established (Mackay 2014; Botstein 2015); we connected it to *C. albicans* genotype-phenotype variation. Second, we showed that the *WOR3-BCR1* relationship could be used for functional gene discovery to define a set of antibiofilm genes. *YWP1* is a known antibiofilm gene (Granger et al. 2005; Sellam et al. 2009; Granger 2012; Granger 2018), but to our knowledge the genes *PGA6*, *DAG7*, and *CWH8* have not been shown to have such a role. The overall relationships that are supported by our data are summarized in Fig. 8.

### Genotype-phenotype variation and shared functions

The rationale for our study was based on the classic idea that 2 genes, gene A and gene B, may have a shared function (Mackay 2014; Botstein 2015). The twist was the suggestion that gene A may be more active in some strains, while gene B is more active in others. Therefore, deletion mutations of either gene may vary in phenotype among different strains. The mutations *bcr1*Δ/Δ, *ume6*Δ/Δ, and *ndt80*Δ/Δ display such variability (Huang et al. 2019; Do et al. 2025; Goerlich and Mitchell 2026). This variability guided our choice of these 3 genes as candidates that may have a shared function with *WOR3*. The finding that *wor3*Δ/Δ *bcr1*Δ/Δ double mutants have severe biofilm defects in all 5 strains tested supports the conclusion that Wor3 and Bcr1 have a shared function in biofilm formation; hence the rationale was useful. Other double mutants (like *ume6*Δ/Δ *wor3*Δ/Δ or *ndt80*Δ/Δ *wor3*Δ/Δ) did not have severe biofilm defects, an indication that there is specificity to the *WOR3-BCR1* genetic interaction.

Our phenotypic analysis argued that Wor3 and Bcr1 have shared targets, and this hypothesis was validated by RNA-seq data. In RPMI+FBS, 188 genes respond to a single *bcr1*Δ/Δ mutation, 84 genes respond to a single *wor3*Δ/Δ mutation, and 20 genes respond to both single mutations, including all 4 antibiofilm genes (Fig. 5; Supplementary Table 2). In RPMI, 124 genes respond to a single *bcr1*Δ/Δ mutation, 740 genes respond to a single *wor3*Δ/Δ mutation, and 56 genes respond to both single mutations, including 3 of the 4 antibiofilm genes (Fig. 5; Supplementary Table 2). These comparisons indicate that Wor3 and Bcr1 do share targets, but only to a limited extent. We can also look at published ChIP-chip data, though growth conditions for Wor3 (Hernday et al. 2013) and Bcr1 (Nobile et al. 2012) datasets were different. Published data show that 252 genes are bound by Bcr1, 119 genes are bound by Wor3, and 49 genes are bound by both, including only *YWP1* among antibiofilm genes (Supplementary Table 2). This comparison also indicates that Wor3 and Bcr1 share a limited set of targets. These levels of overlap suggest that Wor3 and Bcr1 act in convergent pathways, rather than acting in the same signaling pathway.

We often think of transcription factors in terms of the signals to which they respond. Regulation of RNA levels of *WOR3* and *BCR1* have some similarities: both are induced in biofilms (Nobile et al. 2012); both depend upon master regulator Efg1 in most or all *C. albicans* strains tested (Cravener et al. 2023). There are also some differences: *WOR3* is upregulated during hyphal induction under all conditions, whereas *BCR1* responds to hyphal induction in a subset of conditions (Azadmanesh et al. 2017); *WOR3* expression is independent of the master regulator Tec1, whereas *BCR1* expression depends upon Tec1 (Nobile and Mitchell 2005; Nobile et al. 2012). Therefore, Wor3 and Bcr1 both may relay signals associated with biofilm growth and Efg1 activity; Wor3 may be more closely tied to hypha formation; Bcr1 may be more closely tied to Tec1 activity.

### Functional Wor3-Bcr1 target genes

We pursued the idea that upregulated antibiofilm genes may cause the *wor3*Δ/Δ *bcr1*Δ/Δ biofilm defect. Recovery of biofilm formation in the *wor3*Δ/Δ *bcr1*Δ/Δ background by deletion of any of the genes *PGA6, DAG7, CWH8,* and *YWP1* supports the hypothesis that each one is an antibiofilm gene. *YWP1* has well-established antiadhesion effects (Granger et al. 2005; Sellam et al. 2009; Granger 2012; Granger 2018), but to our knowledge the other genes have not been shown to have this role previously. Our studies expand the number of genes in this functional class considerably.

How do these antibiofilm gene products act? Ywp1 is a GPI-linked glycoprotein that is at least in part secreted (Granger et al. 2005). Ywp1 facilitates dispersal during yeast phase growth (Granger 2012) and contributes to masking of underlying β-1,3-glucan in the cell wall (Yang et al. 2022). Pga6 is a predicted GPI-linked cell surface protein that is linked to early stages of biofilm formation through its expression (Fox et al. 2015). Dag7 is secreted in yeast cell culture (Sorgo et al. 2010). Cwh8 is a conserved lipid phosphatase originally characterized in *Saccharomyces cerevisiae* as a dolichol pyrophosphatase (Fernandez et al. 2001). In this context it may be required for biogenesis of Ywp1, Dag7, and Pga6. *C. albicans* Cwh8 is also required for biosynthesis of farnesol (Gutzmann et al. 2025), a quorum-sensing molecule that inhibits *C. albicans* biofilm formation (Ramage et al. 2002). Both roles of Cwh8 may contribute to its antibiofilm function. We hypothesized that Pga6 and Dag7 may form a complex with Ywp1 or support Ywp1 biogenesis. However, our preliminary studies have revealed no defect in Ywp1 secretion in *pga6*Δ/Δ or *dag7*Δ/Δ mutants, nor revealed presence of Pga6- or Dag7-derived peptides associated with secreted Ywp1 (our unpublished results). Detailed analysis of Ywp1 processing and subcellular localization may be necessary to define the molecular relationships among these antibiofilm genes.

Identification of these Wor3-Bcr1 targets expands our view of biofilm-relevant Wor3 and Bcr1 functions. Previous studies of Wor3 (Cravener et al. 2023) and Bcr1 (Nobile and Mitchell 2005; Nobile et al. 2006; Finkel et al. 2012; Nobile et al. 2012; Huang et al. 2019) have focused on their role in activating genes required for biofilm formation. Here we provided evidence that Wor3 and Bcr1 also repress genes that inhibit biofilm formation. An analogous situation exists for biofilm master regulator Brg1. It activates adhesin gene *ALS1* and biofilm positive regulatory gene *FLO8* (Nobile et al. 2012), and also represses 2 transcription factor genes that inhibit biofilm formation, *NRG1* (Lu et al. 2014) and *RME1* (Kim et al. 2024; Kim and Mitchell 2025). It may be useful to screen for antibiofilm genes more broadly, especially because they may define mechanisms to be exploited by antibiofilm therapies.

## Supplementary Material

iyag129_Supplementary_Data

iyag129_Peer_Review_History

## Data Availability

Strains and plasmids are available upon request. The authors affirm that all data necessary for confirming the conclusions of the article are present within the article, figures, and Supplementary materials. RNA-seq data have been deposited in the NCBI Gene Expression Omnibus with accession numbers for (RPMI) GSE319691 and (RPMI+10% FBS) GSE28908. Supplemental material available at *GENETICS* online.
