# Supplementary material for: Shared antibiofilm targets of biofilm regulators Wor3 and Bcr1 in Candida albicans
Source: Genetics. 2026 May 22;233(3):iyag129. doi: 10.1093/genetics/iyag129 (PMC13334108; doi:10.1093/genetics/iyag129)
Supplement: iyag129_Supplementary_Data [file iyag129_supplementary_data.zip › Supplementary_Figure_1_GENETICS-2026-309376.pdf]

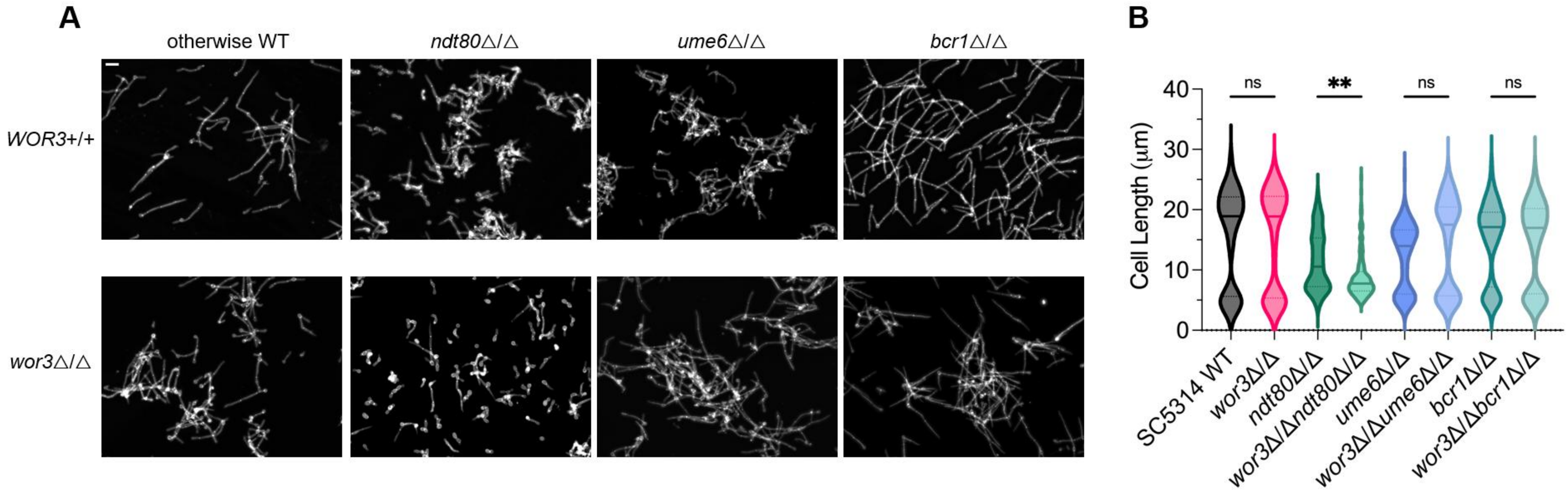

Supplementary Figure 1. Filamentation assays. Strains were grown in prewarmed RPMI medium for 4 hours at 37°C, treated with proteinase K and stained with calcofluor white. (A) Representative fields of view for (top row) wild type, *ndt80*Δ/Δ, *ume6*Δ/Δ, and *bcr1*Δ/Δ, (bottom row) *wor3*Δ/Δ, *wor3*Δ/Δ *ndt80*Δ/Δ, *wor3*Δ/Δ *ume6*Δ/Δ, and *wor3*Δ/Δ *bcr1*Δ/Δ, strains, all in the SC5314 background. The scale bar in the SC5314 WT image is 20 μm and applies to all panels. (B) Cell length measurements. Cell length was measured for the strains in panel A. At least three fields of view were measured. A one-way analysis of variance (Brown-Forsythe test) test was used to determine statistical significance, as indicated by asterisks: *= ≤0.05; **= ≤0.01; ***= ≤0.001; ****= ≤0.0001; ns= not significant.
