# Supplementary material for: Shared antibiofilm targets of biofilm regulators Wor3 and Bcr1 in Candida albicans
Source: Genetics. 2026 May 22;233(3):iyag129. doi: 10.1093/genetics/iyag129 (PMC13334108; doi:10.1093/genetics/iyag129)
Supplement: iyag129_Supplementary_Data [file iyag129_supplementary_data.zip › Supplementary_Figure_2_GENETICS-2026-309376.pdf]

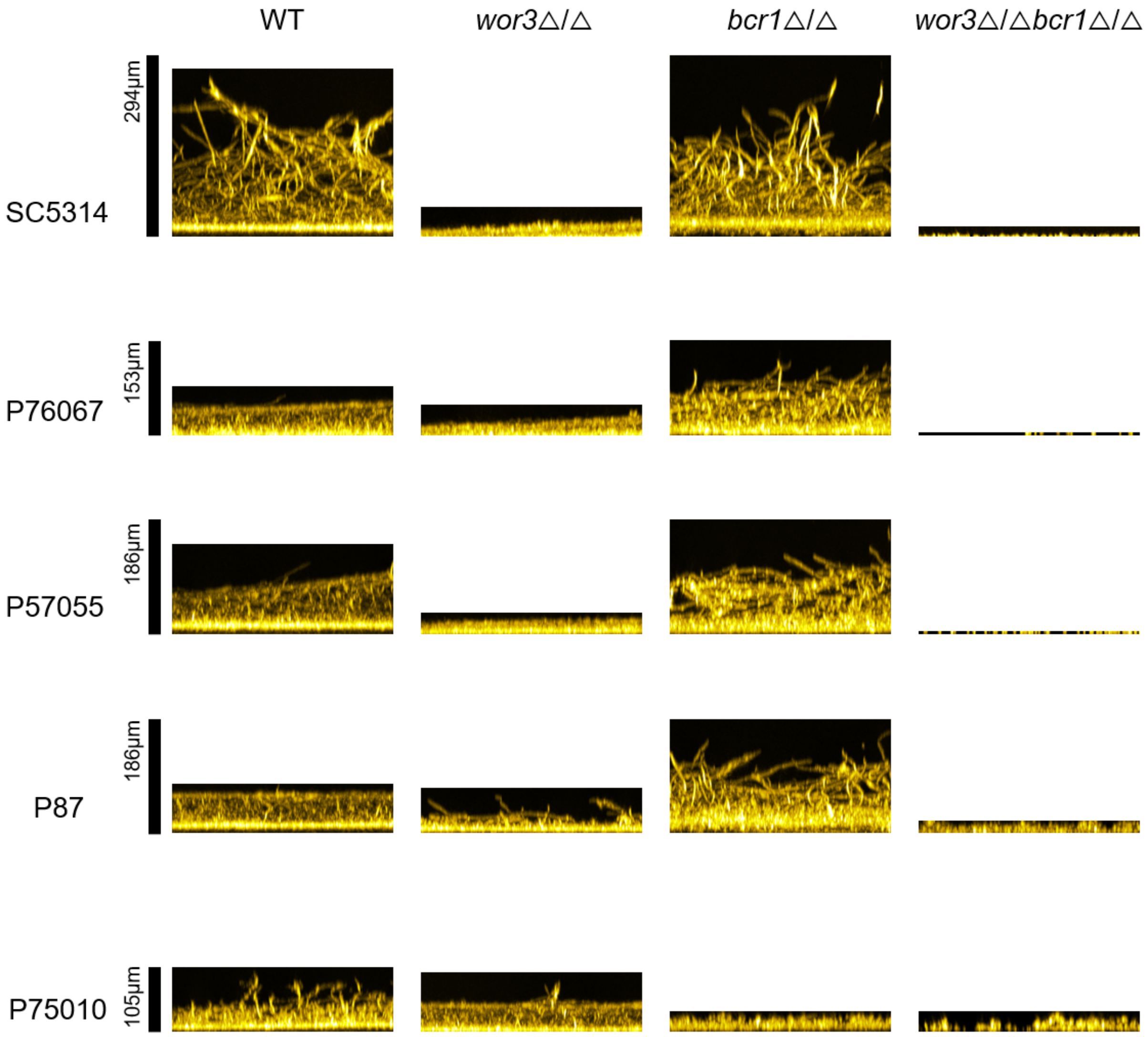


**Supplementary Figure 2.** Biofilm assays in RPMI+FBS medium. *C. albicans* wild-type, *wor3*Δ/Δ, *bcr1*Δ/Δ, *wor3*Δ/Δ *bcr1*Δ/Δ strains from the SC5314, P76067, P57055, P87, and P75010 backgrounds were assayed for biofilm formation in vitro. Strains were grown in RPMI + 10% FBS in a 96 well plate at 37°C for 24 hours. Biofilms were fixed and stain with calcofluor white and imaged using a Keyence BZ-X800E fluorescence microscope. Representative biofilm 96 well plate side projection views were chosen. Scale bars: SC5314, 294µm; P76067, 153µm; P57055, 186µm; P87, 186µm; P75010, 105µm.
