# Supplementary material for: Shared antibiofilm targets of biofilm regulators Wor3 and Bcr1 in Candida albicans
Source: Genetics. 2026 May 22;233(3):iyag129. doi: 10.1093/genetics/iyag129 (PMC13334108; doi:10.1093/genetics/iyag129)
Supplement: iyag129_Supplementary_Data [file iyag129_supplementary_data.zip › Supplementary_Figure_Legends_GENETICS-2026-309376.docx]

**Supplementary Figure Legends**

**Supplementary Figure 1.** Filamentation assays. Strains were grown in prewarmed RPMI medium for 4 hours at 37°C, treated with proteinase K and stained with calcofluor white. (A) Representative fields of view for (top row) wild type, *ndt80*Δ/Δ, *ume6*Δ/Δ, and *bcr1*Δ/Δ, (bottom row) *wor3*Δ/Δ, *wor3*Δ/Δ *ndt80*Δ/Δ, *wor3*Δ/Δ *ume6*Δ/Δ, and *wor3*Δ/Δ *bcr1*Δ/Δ, strains, all in the SC5314 background. The scale bar in the SC5314 WT image is 20 μm and applies to all panels. (B) Cell length measurements. Cell length was measured for the strains in panel A. At least three fields of view were measured. A one-way analysis of variance (Brown-Forsythe test) test was used to determine statistical significance, as indicated by asterisks: *= ≤0.05; **= ≤0.01; ***= ≤0.001; ****= ≤0.0001; ns= not significant.

**Supplementary Figure 2.** Biofilm assays in RPMI+FBS medium. *C. albicans* wild-type, *wor3*Δ/Δ, *bcr1*Δ/Δ, *wor3*Δ/Δ *bcr1*Δ/Δ strains from the SC5314, P76067, P57055, P87, and P75010 backgrounds were assayed for biofilm formation in vitro. Strains were grown in RPMI + 10% FBS in a 96 well plate at 37°C for 24 hours. Biofilms were fixed and stain with calcofluor white and imaged using a Keyence BZ-X800E fluorescence microscope. Representative biofilm 96 well plate side projection views were chosen. Scale bars: SC5314, 294μm; P76067, 153μm; P57055, 186μm; P87, 186μm; P75010, 105μm.
