# Supplementary material for: Shared antibiofilm targets of biofilm regulators Wor3 and Bcr1 in Candida albicans
Source: Genetics. 2026 May 22;233(3):iyag129. doi: 10.1093/genetics/iyag129 (PMC13334108; doi:10.1093/genetics/iyag129)
Supplement: iyag129_Peer_Review_History [file iyag129_peer_review_history.pdf]

GENETICS

Shared anti-biofilm targets of biofilm regulators Wor3 and Bcr1 in Candida albicans

Katharina Goerlich, Norma Solis, Scott Filler, and Aaron Mitchell

NOTE: The reviews and decision letters are unedited and appear as submitted by the reviewers.
In extremely rare instances and as determined by a Senior Editor or the EIC, portions of a review may be redacted. If a review is signed, the reviewer has agreed to no longer remain anonymous.
The review history appears in chronological order.

Review Timeline:

Submission Date:	2026-04-15
Editorial Decision:	2026-05-05
Revision Received:	2026-05-11
Accepted:	2026-05-14

May 5, 2026
RE: GENETICS-2026-309376

Dear Dr. Mitchell:

I am pleased to accept your manuscript titled "Shared anti-biofilm targets of biofilm regulators Wor3 and Bcr1 in Candida albicans" for publication in GENETICS, pending minor revision.

Please submit your revision along with a brief description of how you modified the manuscript in response to the reviewers' concerns and suggestions (which can be viewed at the bottom of this email. I expect you should be able to submit a revised manuscript within 30 days. A suitably revised manuscript will be acceptable for publication; I don't expect to send it out for review.

Prior to uploading your revised manuscript please format it according to GENETICS style and ensure you have all required elements. Author Guidelines are at https://academic.oup.com/genetics/pages/author-guidelines#section-12. These guidelines have been updated with new requirements and your careful attention is required to avoid delays at the source file stage post-acceptance.

When revising the ms., please make an effort to shorten it, because that almost always improves a manuscript. We urge authors to heed the advice of Strunk and White: "omit needless words"1. Follow this link to submit the revised manuscript: Link Not Available

Thank you for submitting this story to Genetics.

Sincerely,

Laura Rusche
Associate Editor
GENETICS

Approved by:
Karen Arndt
Senior Editor
GENETICS

Reviewer comments:
Reviewer #1 :

Biofilms are important contributors to fungal virulence and, in Candida albicans, are governed by a complex regulatory network involving many transcription factors. There is some evidence, by this group and others, that this network differs by environmental condition and even by strain. This manuscript is an investigation of variability in five strains and essentially concludes that the transcription factors Bcr1 and Wor3 have overlapping roles that have been adapted by strain such that Bcr1 is more important in some, and Wor3 in others, but the double bcr1Δ wor3Δ mutant is severely affected in all. Transcriptomics identified differentially expressed genes in the mutants, including several that are biofilm repressors (a question that has received much less attention than the activators) and triple mutants (bcr1Δ wor3Δ repressorΔ) restore biofilm formation to some degree in all the strains, an intellectually satisfying, if somewhat surprising, result.

The genetics of all of this is comprehensive and very clear. This really strengthens the argument that studies of complex phenomena need to be done in multiple strains, because the picture generated by the type strain SC5314 was incomplete. They could actually emphasize this more in the discussion, but otherwise their interpretations are clear. This is a fairly straightforward paper, but well-written and conclusive. The subject is appropriate for Genetics. I have only minor comments.

1. The model at the end is somewhat unsatisfying. It presents a simplified view (as models should do), but implies there are four downstream targets, when there are probably many more, and does not propose anything upstream of the transcription factors. Are they activated by different signals, the same? Are Bcr1 and Wor3 solely repressors of repressors, or is there positive regulation as well. A bit more thought here would be helpful.

2. Scale bars for each row of the lateral biofilm images would be helpful. One presumes that the 182μm bar in figure 7A, as an example, applies to each of the five rows, but it would be nice to see the actual dimensions.

3. The formatting of the gene tables in Fig. 5A and B are different in the table header and colors. Minor, of course, but this would be better if they were the same.

Reviewer #2 :

In this study the authors investigated why the relative importance of several transcriptional regulators of biofilm formation is variable among different C. albicans strains. They hypothesized that this may be due to strain-specific differences in the relative activity of regulators with overlapping functions and that mutants lacking two such regulators would have a defect in biofilm formation in many strains. By generating and analyzing a series of double deletion mutants they found that absence of both Wor3 and Bcr1, which alone usually had a mild effect on biofilm formation, caused a severe biofilm defect in all strains tested. Transcriptional profiling showed that Wor3 and Bcr1 shared a limited set of target genes, several of which encoded cell wall-related proteins and were strongly upregulated in a wor3Δ bcr1Δ double mutant. Strikingly, deletion of almost any of these genes in the double mutant restored biofilm formation, demonstrating that they have anti-biofilm functions that are repressed by Wor3 and Bcr1.
This is a thorough and well performed study that provides novel insights into the regulation of biofilm formation in different C. albicans strains. The manuscript is very well written and was a pleasure to read.

Comments
1) I missed a standard control experiment for the effect of the wor3Δ bcr1Δ double deletion on biofilm formation, at least for the mutants generated in the reference strain SC5314 (Figure 1). Reintroduction of WOR3 should restore wild-type biofilm formation (as in the bcr1Δ single mutant) and reintroduction of BCR1 should improve biofilm formation to the level seen for the wor3Δ single mutant. On the other hand, in a later experiment (the competition experiment shown in Figure 4) two independently constructed double mutants were tested and gave the same results, so the complementation control may be considered less imporant in this case.
2) The authors may add quantitative data to Supplementary Figure 2 (if determined), as in Figures 1B and 2B.

Minor editorial comments
3) Line 124: Either "a wor3Δ mutant exhibited reduced biofilm depth..." or "WOR3 deletion caused reduced biofilm depth..."
4) Line 664: "...in either prewarmed RPMI+10% fetal bovine serum (RPMI+FBS) or RPMI" formally implies that the latter was not prewarmed. Is that true? I assume that both media were prewarmed and suggest to rephrase the sentence.

Reviewer #3 :

This manuscript identifies novel mechanisms regulating biofilm formation in Candida albicans. The results show that the transcription factors Wor3 and Bcr1 have overlapping functions in regulating biofilm formation. This topic is of broad interest on a basic research level as an example of how a large network of transcription factors function. It is also significant since biofilms are one of the major underlying factors in initiating lethal C. albicans infections.

The initial goal of the manuscript was to try to understand why the importance of WOR3 and BCR1 varies in different clinical isolates of C. albicans. The authors tested the hypothesis that their functions overlap by analyzing the effects of wor3 bcr1 double mutations in 5 different clinical isolates. The data support the conclusion that Wor3 and Bcr1 have overlapping functions. This research was also interesting in the way that the authors used a very diverse clinical isolates to see how gene function can vary within a population, rather than focusing on just one selected model strain.

RNAseq analysis of the single and double mutant strains revealed a set of genes that were repressed by Wor3 and Bcr1, including a known anti-biofilm gene YWP1. Targeted analysis of some other repressed genes predicted to encode secreted/cell wall proteins identified three other anti-biofilm genes (DAG7, PGA6 and CWH8). The conclusion that these are novel antibiofilm genes was supported by experiments showing that deletion of these genes restored biofilm formation to a wor3 bcr1 mutant.

Altogether, this is an interesting study of the variation in regulatory pathways for different isolates in a population that provides new insights into biofilm formation. The study of the antibiofilm gene was also interesting as most previous studies have focused on genes that promote biofilm formation.

1. The authors should describe whether known biofilm promoting adhesins were expressed in the RNAseq studies of the wor3, bcr1, and wor3 bcr1 double mutant strains?

2. It would be interesting to know more about the predicted proteins encoded by the anti-biofilm genes (Ywp1, Dag7, Pga6 and Cwh8). They do not seem to be well-studied, but it would be interesting to know if there are any other predictions about their potential roles.

Minor Comments:

- Page 17. Explain what is meant by YFG1.
- Page 17. State more clearly that primers are described more fully in Supplementary Table 2, under the tab for primers.

Associate Editor comments:

Please note that revised manuscript has been restructured to comply with GENETICS formatting instructions (e.g., placing Methods before Results, keeping Abstract <250 words). We used the original submission's format in making editorial changes, which are shown the document comparison file "Manuscript version comparison."

Reviewer comments:
Reviewer #1 :

Biofilms are important contributors to fungal virulence and, in Candida albicans, are governed by a complex regulatory network involving many transcription factors. There is some evidence, by this group and others, that this network differs by environmental condition and even by strain. This manuscript is an investigation of variability in five strains and essentially concludes that the transcription factors Bcr1 and Wor3 have overlapping roles that have been adapted by strain such that Bcr1 is more important in some, and Wor3 in others, but the double bcr1Δ wor3Δ mutant is severely affected in all. Transcriptomics identified differentially expressed genes in the mutants, including several that are biofilm repressors (a question that has received much less attention than the activators) and triple mutants (bcr1Δ wor3Δ repressorΔ) restore biofilm formation to some degree in all the strains, an intellectually satisfying, if somewhat surprising, result.

The genetics of all of this is comprehensive and very clear. This really strengthens the argument that studies of complex phenomena need to be done in multiple strains, because the picture generated by the type strain SC5314 was incomplete. They could actually emphasize this more in the discussion, but otherwise their interpretations are clear. This is a fairly straightforward paper, but well-written and conclusive. The subject is appropriate for Genetics. I have only minor comments.

Sincere thanks for this positive summary.

1. The model at the end is somewhat unsatisfying. It presents a simplified view (as models should do), but implies there are four downstream targets, when there are probably many more, and does not propose anything upstream of the transcription factors. Are they activated by different signals, the same? Are Bcr1 and Wor3 solely repressors of repressors, or is there positive regulation as well. A bit more thought here would be helpful.

We hoped to avoid summarizing the upstream signals in the figure. The reason is that the details of signaling are not well worked out, as described in our Discussion paragraph that begins "We often think of transcription factors in terms of the signals to which they respond." Thus trying to include them in a summary figure would require a lot of explanation that would take us away from the central themes of our study. We also hoped to avoid a broader presentation of Bcr1 and Wor3 targets – both positively and negatively regulated one – because that will dilute out what is unique and interesting (we think!) about our study. But we have to agree with you that the original summary figure was a bit of a letdown. So…

See what you think of our new Figure 8. Here we tried to capture the key points of this specific study: (i) connecting anti-biofilm genes to Wor3 and Bcr1, and (ii) connecting Wor3 and Bcr1 to strain variation. I think that point (ii) is a more novel element in our study, and perhaps capturing it in the figure will inspire other investigators to consider the issue in useful ways.

Other than that, I can only promise that I plan to dedicate fall 2026 to writing a more comprehensive perspective or review article on biofilm regulation in *C. albicans*. One of the important issues I hope to deal with is how stringing together a series of 2-gene epistasis tests – often conducted under different growth conditions and with different output assays – can give a deceptive view of complex regulatory pathways. Hopefully I can make it interesting or, failing that, at least useful.

2. Scale bars for each row of the lateral biofilm images would be helpful. One presumes that the 182µm bar in figure 7A, as an example, applies to each of the five rows, but it would be nice to see the actual dimensions.

We have added scale bars as suggested to Figures 1A, 2A, 7A, and S2. We also reformatted figure 6A to improve resolution.

3. The formatting of the gene tables in Fig. 5A and B are different in the table header and colors. Minor, of course, but this would be better if they were the same.

We fixed it. Thanks so much for calling this to our attention.

Reviewer #2 :

In this study the authors investigated why the relative importance of several transcriptional regulators of biofilm formation is variable among different C. albicans strains. They hypothesized that this may be due to strain-specific differences in the relative activity of regulators with overlapping functions and that mutants lacking two such regulators would have a defect in biofilm formation in many strains. By generating and analyzing a series of double deletion mutants they found that absence of both Wor3 and Bcr1, which alone usually had a mild effect on biofilm formation, caused a severe biofilm defect in all strains tested. Transcriptional profiling showed that Wor3 and Bcr1 shared a limited set of target genes, several of which encoded cell wall-related proteins and were strongly upregulated in a wor3Δ bcr1Δ double mutant. Strikingly, deletion of almost any of these genes in the double mutant restored biofilm formation, demonstrating that they have anti-biofilm functions that are repressed by Wor3 and Bcr1.
This is a thorough and well performed study that provides novel insights into the regulation of biofilm formation in different C. albicans strains. The manuscript is very well written and was a pleasure to read.

Comments

1) I missed a standard control experiment for the effect of the wor3Δ bcr1Δ double deletion on biofilm formation, at least for the mutants generated in the reference strain SC5314 (Figure 1). Reintroduction of WOR3 should restore wild-type biofilm formation (as in the bcr1Δ single mutant) and reintroduction of BCR1 should improve biofilm formation to the level seen for the wor3Δ single mutant. On the other hand, in a later experiment (the competition experiment shown in Figure 4) two independently constructed double mutants were tested and gave the same results, so the complementation control may be considered less imporant in this case.

You raised a good point about complementation. We felt as you said that use of two independent isolates in SC5314, as well as the consistent results in multiple strain backgrounds, provided solid support for our major conclusions.

2) The authors may add quantitative data to Supplementary Figure 2 (if determined), as in Figures 1B and 2B.

I hope you will forgive us for declining to include quantitative data. It is quite time-consuming to calculate biofilm volumes, and the Figure S2 results are intended to show that RPMI+FBS gives similar biofilm assay results to RPMI for the genotypes and strains under study. This in turn is intended to explain for the reader why we looked at both RPMI+FBS and RPMI for RNA-seq, rather than using only RPMI as we did in most of the study.

Minor editorial comments
3) Line 124: Either "a wor3Δ mutant exhibited reduced biofilm depth..." or "WOR3 deletion caused reduced biofilm depth..."

Fixed – thank you!

4) Line 664: "...in either prewarmed RPMI+10% fetal bovine serum (RPMI+FBS) or RPMI" formally implies that the latter was not prewarmed. Is that true? I assume that both media were prewarmed and suggest to rephrase the sentence.

Fixed – thank you! Yes, both media were prewarmed.

Reviewer #3 :

This manuscript identifies novel mechanisms regulating biofilm formation in Candida albicans. The results show that the transcription factors Wor3 and Bcr1 have overlapping functions in regulating biofilm formation. This topic is of broad interest on a basic research level as an example of how a large network of transcription factors function. It is also significant since biofilms are one of the major underlying factors in initiating lethal C. albicans infections.

The initial goal of the manuscript was to try to understand why the importance of WOR3 and BCR1 varies in different clinical isolates of C. albicans. The authors tested the hypothesis that their functions overlap by analyzing the effects of wor3 bcr1 double mutations in 5 different clinical isolates. The data support the conclusion that Wor3 and Bcr1 have overlapping functions. This research was also interesting in the way that the authors used a very diverse clinical isolates to see how gene function can vary within a population, rather than focusing on just one selected model strain.

RNAseq analysis of the single and double mutant strains revealed a set of genes that were repressed by Wor3 and Bcr1, including a known anti-biofilm gene YWP1. Targeted analysis of some other repressed genes predicted to encode secreted/cell wall proteins identified three other anti-biofilm genes (DAG7, PGA6 and CWH8). The conclusion that these are novel antibiofilm genes was supported by experiments showing that deletion of these genes restored biofilm formation to a wor3 bcr1 mutant.

Altogether, this is an interesting study of the variation in regulatory pathways for different isolates in a population that provides new insights into biofilm formation. The study of the antibiofilm gene was also interesting as most previous studies have focused on genes that promote biofilm formation.

Thank you for this detailed and supportive summary!

1. The authors should describe whether known biofilm promoting adhesins were expressed in the RNAseq studies of the wor3, bcr1, and wor3 bcr1 double mutant strains?

We have added two sentences to the Results section on RNA-seq outcomes:
“The adhesin gene *ALS1* was downregulated in *wor3*Δ/Δ *bcr1*Δ/Δ double mutant vs wild type comparison in RPMI+FBS (Figure 5B); however, this gene expression change seemed unlikely to account for the *wor3*Δ/Δ *bcr1*Δ/Δ double mutant phenotype. The reason is that we have seen no impact of *ALS1* and the related adhesin gene *ALS3* on biofilm formation in RPMI medium (our unpublished results)”

We hope to avoid more detail about adhesins because they will distract from the major and more novel issues of strain variation and anti-biofilm genes.

2. It would be interesting to know more about the predicted proteins encoded by the anti-biofilm genes (Ywp1, Dag7, Pga6 and Cwh8). They do not seem to be well-studied, but it would be interesting to know if there are any other predictions about their potential roles.

Good idea! We actually spent a lot of time on such studies but did not get any compelling results. In any case, we have expanded a Discussion paragraph on this topic, as follows:
“How do these anti-biofilm gene products act? Ywp1 is a GPI-linked glycoprotein that is at least in part secreted [28]. Ywp1 facilitates dispersal during yeast phase growth [30]

and contributes to masking of underlying β-1,3-glucan in the cell wall [37]. Pga6 is a predicted GPI-linked cell surface protein that is linked to early stages of biofilm formation through its expression [9]. Dag7 is secreted in yeast cell cultures [38]. Cwh8 is a conserved lipid phosphatase originally characterized in *Saccharomyces cerevisiae* as a dolichol pyrophosphatase [39]. In this context it may be required for biogenesis of Ywp1, Dag7, and Pga6. *C. albicans* Cwh8 is also required for biosynthesis of farnesol [40], a quorum-sensing molecule that inhibits *C. albicans* biofilm formation [41]. Both roles of Cwh8 may contribute to its anti-biofilm function. We hypothesized that Pga6 and Dag7 may form a complex with Ywp1 or support Ywp1 biogenesis. However, our preliminary studies have revealed no defect in Ywp1 secretion in *pga6*Δ/Δ or *dag7*Δ/Δ mutants, nor revealed presence of Pga6- or Dag7-derived peptides associated with secreted Ywp1 (our unpublished results). Detailed analysis of Ywp1 processing and subcellular localization may be necessary to define the molecular relationships among these antibiofilm genes."

Minor Comments:
• Page 17. Explain what is meant by YFG1.

We modified the sentence; is this OK?
"Homozygous knockout mutants were made by targeting each respective gene, which we refer to generically as "*YFG1* (Your Favorite Gene 1)" with 1 μg Cas9 DNA cassette, 1 μg sgRNA DNA cassette, 3 μg of *YFG1* deletion cassette."

• Page 17. State more clearly that primers are described more fully in Supplementary Table 2, under the tab for primers.

In that same Methods section we now say:
"(Please see supplementary table 2 for details about plasmids and primers, each in a separate tab.)" Please note that the supplementary table numbers had to be reversed in the final version because Methods precedes Results. Hence in the final version S1 is strains/primers/plasmids and S2 is RNA-seq.

May 13, 2026
RE: GENETICS-2026-309376R1

Dr. Aaron P. Mitchell
University of Georgia
Department of Microbiology
527 Biological Sciences Building
Athens, Georgia 30602

Dear Dr. Mitchell:

Congratulations, your manuscript titled "Shared anti-biofilm targets of biofilm regulators Wor3 and Bcr1 in Candida albicans" is accepted for publication in GENETICS! Many thanks for submitting your research to the journal.

To Proceed to Publication:
1. Format your article according to GENETICS style: https://academic.oup.com/genetics/pages/author-guidelines

2. Ensure that you comply with data and community resource citation guidelines:
https://academic.oup.com/genetics/pages/author-guidelines#section-5-9-2

3. Upload your final files at https://genetics.msubmit.net

4. Add oupsupport@scipris.com and genetics.oup@novatechset.com (or the domains @scipris.com and @novatechset.com) to your email program's "safe senders" list. You will be contacted by both at various points during the production process.

Notes:
- Your currently-accepted manuscript (unedited, as submitted, reviewed, and accepted) will be published at GENETICS and deposited into PubMed as an Advance Access article. Notify sourcefiles@thegsajournals.org before signing your license if you do not wish to publish your article via Advance Access.

- We invite you to submit an original color figure related to your paper for consideration as cover art. Please email your submission to the editorial office or upload it with your final files. You can submit a small-sized image for evaluation, and if selected, the final image must be a TIFF file 2513px wide by 3263px high (8.375 by 10.875 inches; resolution of 600ppi). Please avoid graphs and small type.

- After files are sent to Oxford University Press we use SciPris to manage article licensing and payment. If you do not have a SciPris account, you will receive an email from no-reply@scipris.com to sign up to use Oxford University Press' author portal. After logging in, follow the online instructions to sign your license and arrange any payment due.

If you have any questions or encounter any problems while uploading your accepted manuscript files, please email the editorial office at sourcefiles@thegsajournals.org.

Sincerely,

Laura Rusche
Associate Editor
GENETICS

Approved by:
Karen Arndt
Senior Editor
GENETICS

Review comments (if applicable):
